# Supplementary material for: Multi-omics Analyses Provide Insight into the Biosynthesis Pathways of Fucoxanthin in Isochrysis galbana
Source: Genomics Proteomics Bioinformatics. 2022 Aug 13;20(6):1138–53. doi: 10.1016/j.gpb.2022.05.010 (PMC10225490; doi:10.1016/j.gpb.2022.05.010)
Supplement: Supplementary Table S1 — Sequencing data used for I. galbana LG007 genome construction [file mmc1.docx]

**Table S1 Sequencing data used for** ***I*. *galbana* LG007 genome construction**

| **Library**  **resource** | **Sequencing platform** | **Insert size** | **Clean data (Gb)** | **Sequence coverage (X)** | **Use of the data** |
| --- | --- | --- | --- | --- | --- |
| Genome | Illumina HiSeq X Ten | 250 bp | 8.92 | 96 | Genome estimation and polishing |
| Genome | PacBio Sequel | 20 kb | 15.53 | 166 | Genome assembly |
| Hi-C | Illumina HiSeq X Ten | 250 bp | 12.35 | 137 | Chromosome construction |
| Transcriptome | Illumina HiSeq X Ten | 0.6–3 kb | 307.77 | - | Difference analysis and annotation |

*Note*: Hi-C, high-throughput chromatin conformation capture.
