# Supplementary material for: Multi-omics Analyses Provide Insight into the Biosynthesis Pathways of Fucoxanthin in Isochrysis galbana
Source: Genomics Proteomics Bioinformatics. 2022 Aug 13;20(6):1138–53. doi: 10.1016/j.gpb.2022.05.010 (PMC10225490; doi:10.1016/j.gpb.2022.05.010)
Supplement: Supplementary Table S2 — Assembly statistics for nuclear genome [file mmc2.docx]

**Table S2 Assembly statistics for nuclear genome**

| **Items** | **Statistics** |
| --- | --- |
| Estimated genome size (Mb) | 92.59 |
| Genomic G+C content | 58.44% |
| Number of assembled scaffolds | 353 |
| Number of scaffolds > 2 kb | 353 |
| Min length (bp) | 5065 |
| Max length (bp) | 2,925,745 |
| Average length (bp) | 262,298 |
| Scaffolds N50 (kb) | 666.7 |
| Predicted protein-coding genes | 14,900 |
| Average coding sequence length (bp) | 1428 |
| Average transcript length (bp) | 1764 |
| Average gene length (bp) | 1789 |
| Chromosomal assembled N50 (Mb) | 6.99 |
