# Supplementary material for: Multi-omics Analyses Provide Insight into the Biosynthesis Pathways of Fucoxanthin in Isochrysis galbana
Source: Genomics Proteomics Bioinformatics. 2022 Aug 13;20(6):1138–53. doi: 10.1016/j.gpb.2022.05.010 (PMC10225490; doi:10.1016/j.gpb.2022.05.010)
Supplement: Supplementary Table S3 — Assessment of the completeness of the I. galbana LG007 genome assembly by BUSCO [file mmc3.docx]

**Table S3 Assessment of the completeness of the *I*. *galbana* LG007 genome assembly by BUSCO**

| **Type** | **Number** | **Percent (%)** |
| --- | --- | --- |
| Complete BUSCOs (C) | 254 | 83.8 |
| Complete and single-copy BUSCOs (S) | 248 | 81.8 |
| Complete and duplicated BUSCOs (D) | 6 | 2.0 |
| Fragmented BUSCOs (F) | 13 | 4.3 |
| Missing BUSCOs (M) | 36 | 11.9 |
| Total BUSCO groups searched | 303 | 100 |

*Note*: BUSCO, Benchmarking Universal Single-Copy Orthologs.
