# Supplementary material for: Multi-omics Analyses Provide Insight into the Biosynthesis Pathways of Fucoxanthin in Isochrysis galbana
Source: Genomics Proteomics Bioinformatics. 2022 Aug 13;20(6):1138–53. doi: 10.1016/j.gpb.2022.05.010 (PMC10225490; doi:10.1016/j.gpb.2022.05.010)
Supplement: Supplementary Table S4 — Genomic resequencing alignment rate and coverage assessment [file mmc4.docx]

**Table S4 Genomic resequencing alignment rate and coverage assessment**

| **Items** | **Statistics** |
| --- | --- |
| Unmapped bases (Mb) | 144.6 |
| Mapped bases (Mb) | 887.5 |
| Map rate (%) | 98.4 |
| Genome length (Mb) | 93 |
| Mean depth | 89 |
| Coverage rate (%) | 97.6 |
