# Supplementary material for: Multi-omics Analyses Provide Insight into the Biosynthesis Pathways of Fucoxanthin in Isochrysis galbana
Source: Genomics Proteomics Bioinformatics. 2022 Aug 13;20(6):1138–53. doi: 10.1016/j.gpb.2022.05.010 (PMC10225490; doi:10.1016/j.gpb.2022.05.010)
Supplement: Supplementary Table S5 — Pacbio data alignment rate [file mmc5.docx]

**Table S5 Pacbio data alignment rate**

| **Items** | **Statistics** |
| --- | --- |
| QC-passed reads | 288,211 |
| Duplicates | 0 |
| Mapped reads | 287,594 |
| Mapping rate (%) | 99.78 |

*Note*: QC, quality control.
