# Supplementary material for: Multi-omics Analyses Provide Insight into the Biosynthesis Pathways of Fucoxanthin in Isochrysis galbana
Source: Genomics Proteomics Bioinformatics. 2022 Aug 13;20(6):1138–53. doi: 10.1016/j.gpb.2022.05.010 (PMC10225490; doi:10.1016/j.gpb.2022.05.010)
Supplement: Supplementary Table S6 — Pseudomolecule length statistics after Hi-C assisted assembly [file mmc6.docx]

**Table S6 Pseudomolecule length statistics after Hi-C assisted assembly**

| **Pseudomolecule** | **Length (bp)** |
| --- | --- |
| Chr1 | 11,908,665 |
| Chr2 | 10,401,026 |
| Chr3 | 7,919,487 |
| Chr4 | 7,664,558 |
| Chr5 | 7,036,706 |
| Chr6 | 6,994,209 |
| Chr7 | 6,185,998 |
| Chr8 | 5,984,543 |
| Chr9 | 5,214,086 |
| Chr10 | 4,726,453 |
| Chr11 | 4,149,466 |
| Chr12 | 3,870,612 |
| Chr13 | 3,512,008 |
| Chr14 | 3,046,428 |
| Chr15 | 2,341,788 |
| Total anchored | 90,956,033 |
| Unanchored | 1,777,877 |
