# Supplementary material for: Multi-omics Analyses Provide Insight into the Biosynthesis Pathways of Fucoxanthin in Isochrysis galbana
Source: Genomics Proteomics Bioinformatics. 2022 Aug 13;20(6):1138–53. doi: 10.1016/j.gpb.2022.05.010 (PMC10225490; doi:10.1016/j.gpb.2022.05.010)
Supplement: Supplementary Table S7 — Quality assembly statistics of the I. galbana after the Hi-C data based pseudo-chromosome assembly [file mmc7.docx]

**Table S7 Quality assembly statistics of the *I*. *galbana* after the Hi-C data based pseudo-chromosome assembly**

| **Items** | **Canu** | |  | **Hi-C** | |
| --- | --- | --- | --- | --- | --- |
|  | **Contig_len (Mb)** | **Contig_number** |  | **Contig_len (Mb)** | **Contig_number** |
| Total | 92.59 | 353 |  | 92.64 | 142 |
| Max | 2.92 | - |  | 11.91 | - |
| Number >= 2 kb | - | 353 |  | - | 78 |
| N50 | 0.66 | - |  | 6.99 | - |
