# Supplementary material for: Multi-omics Analyses Provide Insight into the Biosynthesis Pathways of Fucoxanthin in Isochrysis galbana
Source: Genomics Proteomics Bioinformatics. 2022 Aug 13;20(6):1138–53. doi: 10.1016/j.gpb.2022.05.010 (PMC10225490; doi:10.1016/j.gpb.2022.05.010)
Supplement: Supplementary Table S8 — Statistics for gene, exon, CDS and introne in I. galbana LG007 genome [file mmc8.docx]

**Table S8 Statistics for gene, exon, CDS, and introne in *I*. *galbana* LG007 genome**

|  | **Gene stat** | **Exons stat** | **CDS stat** | **Introne stat** |
| --- | --- | --- | --- | --- |
| Total length | 26,666,990 | 21,283,343 | 21,283,343 | 5,383,647 |
| Total number | 14,900 | 43,481 | 14,900 | 28,581 |
| Average length | 1789.73 | 489.49 | 1428.41 | 188.36 |

*Note*: CDS, coding sequence.
