# Supplementary material for: Multi-omics Analyses Provide Insight into the Biosynthesis Pathways of Fucoxanthin in Isochrysis galbana
Source: Genomics Proteomics Bioinformatics. 2022 Aug 13;20(6):1138–53. doi: 10.1016/j.gpb.2022.05.010 (PMC10225490; doi:10.1016/j.gpb.2022.05.010)
Supplement: Supplementary Table S9 — Annotation statistics for the I. galbana LG007 genome [file mmc9.docx]

**Table S9 Annotation statistics for the *I*. *galbana* LG007 genome**

| **Annotation statistics for nuclear genome** | **Number** | **Percent (%)** |
| --- | --- | --- |
| Total protein | 14,900 |  |
| NR | 12,469 | 83.68 |
| eggNOG | 9161 | 61.48 |
| GO | 4977 | 33.40 |
| COG | 9161 | 61.48 |
| KEGG | 3773 | 25.32 |
| At least in one database | 12,500 | 83.89 |

*Note*: NR, Non-Redundant; GO, Gene Ontology; COG, Clusters of Orthologous Groups; KEGG, Kyoto Encyclopedia of Genes and Genomes.
