# Supplementary material for: Multi-omics Analyses Provide Insight into the Biosynthesis Pathways of Fucoxanthin in Isochrysis galbana
Source: Genomics Proteomics Bioinformatics. 2022 Aug 13;20(6):1138–53. doi: 10.1016/j.gpb.2022.05.010 (PMC10225490; doi:10.1016/j.gpb.2022.05.010)
Supplement: Supplementary Table S10 — Transcription factors [file mmc10.docx]

**Table S10 Transcription factors**

| **Number** | **Classify** |
| --- | --- |
| 198 | Protein kinase family protein |
| 55 | Heat shock protein |
| 49 | WD-40 repeat family protein / zfwd4 protein |
| 44 | MYB domain protein 3r-3 |
| 23 | Pentatricopeptide repeat-containing protein |
| 13 | Calmodulin-binding transcription activator protein |
| 11 | CCCH-type zinc finger protein with ARM repeat domain |
| 7 | MYB-like DNA-binding domain |
| 7 | Zinc finger (C2H2 type) family protein / transcription factor jumonji family protein |
| 6 | Homeodomain-like superfamily protein |
| 6 | Ethylene induced calmodulin binding protein |
| 6 | Pathogenesis related homeodomain protein A |
| 3 | GATA transcription factor 15 |
| 3 | E2F transcription factor |
| 2 | Winged-helix DNA-binding transcription factor family protein |
| 2 | Plant-specific transcription factor YABBY family protein |
| 1 | AP2/B3-like transcriptional factor family protein |
| 1 | HD-ZIP IV family of homeobox-leucine zipper protein with lipid-binding START domain |
| 1 | Homeobox-leucine zipper protein 4 (HB-4) / HD-ZIP protein |
| 1 | K-box region and MADS-box transcription factor family protein |

*Note*: WD, beta-transducin; ZFWD, Zinc finger WD40; MYB, V-myb avian myeloblastosis viral oncogene homolog; ARM, Armadillo; GATA, Conserved WGATAR (W = T or A; R = G or A) motifs involved in erythroid-specific gene expressionin vertebrates; E2F, Early region 2 binding factor; YABBY, C2C2 zinc finger-like domain towards the amino terminus and a helix-loop-helix; AP2/B3, A AP2 family proteins contain a B3 domain; HD-ZIP, Homeobox-leucine zipper; MADS-box, A DNA binding domain of 58 amino acids that binds DNA at consensus recognition sequences known as CArG boxes [CC(A/T)6GG].

.
