# Supplementary material for: Multi-omics Analyses Provide Insight into the Biosynthesis Pathways of Fucoxanthin in Isochrysis galbana
Source: Genomics Proteomics Bioinformatics. 2022 Aug 13;20(6):1138–53. doi: 10.1016/j.gpb.2022.05.010 (PMC10225490; doi:10.1016/j.gpb.2022.05.010)
Supplement: Supplementary Table S11 — Statistical analysis of non-coding RNAs in I. galbana LG007 genome [file mmc11.docx]

**Table S11 Statistical analysis of** **non-coding RNAs in *I*. *galbana* LG007 genome**

| **ncRNA items** | **Number** |
| --- | --- |
| tRNA | 95 |
| rRNA | 58 |
| snRNA | 4 |

*Note*: ncRNA, non-coding RNA; tRNA, transfer RNA; rRNA, ribosomal RNA; snRNA, small nuclear RNA.
