# Supplementary material for: Multi-omics Analyses Provide Insight into the Biosynthesis Pathways of Fucoxanthin in Isochrysis galbana
Source: Genomics Proteomics Bioinformatics. 2022 Aug 13;20(6):1138–53. doi: 10.1016/j.gpb.2022.05.010 (PMC10225490; doi:10.1016/j.gpb.2022.05.010)
Supplement: Supplementary Table S12 — Repetitive element annotations in the I. galbana LG007 [file mmc12.docx]

**Table S12 Repetitive element annotations in the *I*. *galbana* LG007**

|  | **No. of TEs** | **Length (bp)** | **% of TEs** | **% of genome** |
| --- | --- | --- | --- | --- |
| Total repeat fraction | 161,907 | 43,349,564 | 100 | 46.82 |
| Class I: retroelement | 73,047 | 31,195,044 | 71.96 | 33.69 |
| LTR retrotransposon | 27,946 | 14,219,065 | 32.8 | 15.36 |
| Ty1/Copia | 1992 | 998,417 | 2.3 | 1.08 |
| Ty3/Gypsy | 4491 | 4,191,464 | 9.67 | 4.53 |
| Other | 21,463 | 9,029,184 | 20.83 | 9.75 |
| non-LTR retrotransposon | 26,756 | 11,607,249 | 26.78 | 12.54 |
| LINE | 21,408 | 10,762,090 | 24.83 | 11.62 |
| SINE | 5348 | 845,159 | 1.95 | 0.91 |
| unclassified retroelement | 18,345 | 5,368,730 | 12.38 | 5.8 |
| Class II: DNA transposon | 33,472 | 7,828,746 | 18.06 | 8.46 |
| CMC | 1176 | 203,681 | 0.47 | 0.22 |
| hAT | 4039 | 1,494,270 | 3.45 | 1.61 |
| Mutator | 629 | 121,730 | 0.28 | 0.13 |
| Tc1/Mariner | 180 | 71,173 | 0.16 | 0.08 |
| PIF/Harbinger | 242 | 98,683 | 0.23 | 0.11 |
| Other | 27,026 | 5,768,036 | 13.31 | 6.23 |
| Helitron | 69 | 12,880 | 0.03 | 0.01 |
| Tandem repeats | 43,633 | 3,981,448 | 9.18 | 4.3 |
| Unkown | 2891 | 1,025,862 | 2.37 | 1.11 |

*Note*: TEs, transcription factors; LTR, long terminal repeat; LINE, long interspersed nuclear elements; SINE, short interspersed nuclear elements; CMC, CACTA nMITE; hAT, hobo, activatorandtam.
