# Supplementary material for: Multi-omics Analyses Provide Insight into the Biosynthesis Pathways of Fucoxanthin in Isochrysis galbana
Source: Genomics Proteomics Bioinformatics. 2022 Aug 13;20(6):1138–53. doi: 10.1016/j.gpb.2022.05.010 (PMC10225490; doi:10.1016/j.gpb.2022.05.010)
Supplement: Supplementary Table S19 — Differentially expressed genes in the comparison of C7d vs. T7d by transcriptome data [file mmc19.docx]

**Table S19 Differentially expressed genes in the comparison of C7d *vs*. T7d by transcriptome data**

| **Gene_id** | **log2FC** | **lfcSE** | **stat** | ***P* value** | ***P* adj** |
| --- | --- | --- | --- | --- | --- |
| IZ000921 | -0.618228045 | 0.28330285 | -2.182216116 | 0.029093586 | 0.128349004 |
| IZ000920 | -0.862878253 | 0.395637297 | -2.180983083 | 0.029184668 | 0.128651816 |
| IZ010869 | -0.019082564 | 0.513613945 | -0.037153517 | 0.970362601 | 0.987349924 |
| IZ011827 | -0.011840673 | 0.34486052 | -0.034334672 | 0.972610277 | 0.98799762 |
| IZ010564 | -0.772164862 | 0.382123114 | -2.02072273 | 0.043308475 | 0.165837741 |
| IZ003505 | -0.300845842 | 0.657648064 | -0.4574572 | 0.647342459 | 0.817137655 |
| IZ008009 | -0.46717575 | 0.49530133 | -0.943215215 | 0.345570833 | 0.581678454 |
| IZ010260 | -0.654490375 | 0.30151432 | -2.170677585 | 0.029955553 | 0.130988806 |
| IZ006832 | 0.358276546 | 0.41590108 | 0.861446541 | 0.388992153 | 0.620149672 |
| IZ012322 | -1.041756606 | 0.374929781 | -2.7785379 | 0.005460414 | 0.042540685 |
| IZ000195 | -1.812689526 | 1.357545286 | -1.335270023 | 0.181787986 | 0.402025948 |
| IZ002967 | 0.095797365 | 0.520894459 | 0.183909357 | 0.854084564 | 0.935664555 |
| IZ007183 | 0.129312115 | 0.50400017 | 0.256571569 | 0.797509525 | 0.907113004 |
| IZ009688 | 1.78319659 | 0.298305616 | 5.97775065 | 2.26E-09 | 3.38E-07 |
| IZ001744 | -1.692288335 | 0.47018672 | -3.599183607 | 0.000319218 | 0.005373199 |
| IZ003288 | -0.726128197 | 0.452481446 | -1.604768998 | 0.108544651 | 0.294645988 |
| IZ000291 | 0.254309903 | 0.288428783 | 0.881707784 | 0.377934851 | 0.610612329 |
| IZ013688 | 1.393393923 | 0.378958137 | 3.67690725 | 0.000236079 | 0.004268348 |
| IZ013190 | -0.215916559 | 0.283158015 | -0.762530274 | 0.445743582 | 0.667805133 |
| IZ002979 | 2.110430225 | 0.414719263 | 5.088816496 | 3.60E-07 | 2.74E-05 |
| IZ005918 | -2.151611413 | 0.423190094 | -5.084266964 | 3.69E-07 | 2.76E-05 |
| IZ001188 | 0.163039038 | 0.373436404 | 0.436591174 | 0.662407864 | 0.827300281 |
| IZ008332 | 1.281168966 | 0.461675097 | 2.775044558 | 0.005519415 | 0.0427694 |
| IZ007195 | -1.15478264 | 0.575781124 | -2.005593084 | 0.04489968 | 0.169885644 |
| IZ006149 | 1.375698459 | 0.302553454 | 4.546960017 | 5.44E-06 | 0.000239136 |
| IZ007042 | -0.637016402 | 0.29061089 | -2.191990816 | 0.02838017 | 0.126337578 |
| IZ007738 | 0.905071091 | 0.322767895 | 2.804092678 | 0.00504584 | 0.040526097 |
| IZ012603 | 1.494212381 | 0.284789023 | 5.246734463 | 1.55E-07 | 1.38E-05 |
| IZ007325 | -0.076755196 | 0.329380162 | -0.233029202 | 0.81573873 | 0.916852587 |
| IZ011107 | -0.545539022 | 0.307710975 | -1.772894262 | 0.07624623 | 0.237586114 |
| IZ005291 | -0.3532401 | 0.314010222 | -1.124931851 | 0.260617914 | 0.493797145 |
| IZ003473 | -0.090417255 | 0.376020193 | -0.240458509 | 0.809974825 | 0.913979253 |
| IZ004535 | 1.205586609 | 0.407258941 | 2.960245895 | 0.003073936 | 0.028450653 |
| IZ008735 | 0.616690163 | 0.304361504 | 2.026176621 | 0.042746689 | 0.16426549 |
| IZ009303 | 0.99199501 | 0.317468966 | 3.124699155 | 0.00177987 | 0.019397025 |
| IZ007092 | -1.657513516 | 0.276271983 | -5.999571508 | 1.99E-09 | 1.32E-07 |
| IZ013676 | 1.152823461 | 0.319632713 | 3.606713001 | 0.0003101 | 0.005271705 |

*Note*: C7d, Control group at 7 day; T7d, Treated group at 7 day; FC, Fold change; lfcSE, The standard error estimate for the log2 fold change estimate.
