# Supplementary material for: Multi-omics Analyses Provide Insight into the Biosynthesis Pathways of Fucoxanthin in Isochrysis galbana
Source: Genomics Proteomics Bioinformatics. 2022 Aug 13;20(6):1138–53. doi: 10.1016/j.gpb.2022.05.010 (PMC10225490; doi:10.1016/j.gpb.2022.05.010)
Supplement: Supplementary Table S20 — Raw data of carotenoid compound content detected by HPLC [file mmc20.docx]

**Table S20 Raw data of carotenoids compound content detected by HPLC**

| **Compound** | **7d-W1** | **7d-W2** | **7d-W3** | **7d-G1** | **7d-G2** | **7d-G3** | ***P* value** | **FC** | **Log2FC** |
| --- | --- | --- | --- | --- | --- | --- | --- | --- | --- |
| β-Carotene | 202.74 | 251.97 | 296.51 | 439.63 | 393.78 | 397.05 | 0.0129 | 1.6379 | 0.7119 |
| ε-Carotene | 0.32 | 0.45 | 0.64 | 1.18 | 1.07 | 0.99 | 0.0088 | 2.2854 | 1.1925 |
| Lutein myristate | 0.07 | 0.08 | 0.07 | 0.19 | 0.08 | 0.07 | 0.3977 | 1.5650 | 0.6461 |
| Violaxanthin laurate | 0.02 | 0.03 | 0.04 | 0.08 | 0.10 | 0.11 | 0.0031 | 3.5034 | 1.8087 |
| Violaxanthin myristate | 13.05 | 11.83 | 11.55 | 43.48 | 24.11 | 26.54 | 0.0865 | 2.5839 | 1.3695 |
| Zeaxanthin myristoleate | 0.04 | 0.05 | 0.05 | N/A | N/A | N/A | N/A | N/A | N/A |
| Zeaxanthin palmitate | 3.50 | 3.11 | 2.34 | 0.85 | 1.18 | 1.31 | 0.0201 | 0.3740 | -1.4189 |
| Antheraxanthin | 0.26 | 0.43 | 0.50 | 0.98 | 1.19 | 1.24 | 0.0026 | 2.8554 | 1.5137 |
| Apocarotenal | 0.05 | 0.03 | 0.04 | 0.08 | 0.06 | 0.06 | 0.0641 | 1.5979 | 0.6762 |
| Canthaxanthin | 0.18 | 0.11 | 0.11 | 0.26 | 0.25 | 0.21 | 0.0254 | 1.8273 | 0.8697 |
| Capsanthin | 0.10 | 0.11 | 0.10 | 0.43 | 0.19 | 0.18 | 0.1822 | 2.5528 | 1.3521 |
| Echinenone | 35.18 | 24.02 | 21.73 | 45.79 | 32.51 | 33.32 | 0.1622 | 1.3792 | 0.4639 |
| Zeaxanthin | 8.35 | 7.30 | 12.04 | 28.16 | 22.62 | 23.14 | 0.0029 | 2.6692 | 1.4164 |
| β-Cryptoxanthin | 12.38 | 7.28 | 5.99 | 14.47 | 9.33 | 9.52 | 0.3791 | 1.2983 | 0.3766 |
| Fucoxanthin | 2.83 | 2.12 | 2.75 | 5.28 | 5.49 | 6.01 | 0.0091 | 2.1476 | 1.1028 |

*Note*: HPLC, high performance liquid chromatography; 7d-W, cultivate for 7d with white light; 7d-G, cultivate for 7d with green light.
