# Supplementary material for: Multi-omics Analyses Provide Insight into the Biosynthesis Pathways of Fucoxanthin in Isochrysis galbana
Source: Genomics Proteomics Bioinformatics. 2022 Aug 13;20(6):1138–53. doi: 10.1016/j.gpb.2022.05.010 (PMC10225490; doi:10.1016/j.gpb.2022.05.010)
Supplement: Supplementary Table S21 — Differentially accumulated carotenoids compounds in the comparison of 7d-W vs. 7d-G by targeted metabolomics (n = 3) [file mmc21.docx]

**Table S21 Differentially accumulated carotenoids compounds in the comparison of 7d-W *vs*. 7d-G by targeted metabolomics (n = 3)**

| **Compound** | **Class** | ***P* value** | **FC** | **Log_2_ FC** |  |
| --- | --- | --- | --- | --- | --- |
| ε-Carotene | carotenes | 0.0088 | 2.2854 | 1.1925 | up |
| violaxanthin laurate | carotenoid esters | 0.0031 | 3.5034 | 1.8087 | up |
| violaxanthin myristate | carotenoid esters | 0.0087 | 2.5839 | 1.3695 | up |
| zeaxanthin palmitate | carotenoid esters | 0.0201 | 0.3740 | -1.4189 | down |
| antheraxanthin | xanthophylls | 0.0026 | 2.8554 | 1.5137 | up |
| capsanthin | xanthophylls | 0.0018 | 2.5528 | 1.3521 | up |
| zeaxanthin | xanthophylls | 0.0029 | 2.6692 | 1.4164 | up |
| fucoxanthin | xanthophylls | 0.0091 | 2.1476 | 1.1028 | up |
