# Supplementary material for: Multi-omics Analyses Provide Insight into the Biosynthesis Pathways of Fucoxanthin in Isochrysis galbana
Source: Genomics Proteomics Bioinformatics. 2022 Aug 13;20(6):1138–53. doi: 10.1016/j.gpb.2022.05.010 (PMC10225490; doi:10.1016/j.gpb.2022.05.010)
Supplement: Supplementary Table S22 — Statistical analysis of transcriptome data [file mmc22.docx]

**Table S22 Statistical analysis of transcriptome data**

| **Sample** | **Repeat** | **Total_reads** | **Total_bases** | **GC_content** | **Q20** | **Q30** |
| --- | --- | --- | --- | --- | --- | --- |
| C9d | C_9_r1 | 67,821,102 | 10,173,165,300 | 60.12% | 96.11% | 90.83% |
|  | C_9_r2 | 74,208,330 | 11,131,249,500 | 60.33% | 96.10% | 90.82% |
|  | C_9_r3 | 83,696,476 | 12,554,471,400 | 60.12% | 96.14% | 90.85% |
| C7d | C_7_r1 | 108,882,342 | 16,332,351,300 | 60.70% | 95.50% | 89.52% |
|  | C_7_r2 | 90,029,112 | 13,504,366,800 | 60.14% | 95.83% | 90.21% |
|  | C_7_r3 | 75,430,952 | 11,314,642,800 | 60.14% | 95.97% | 90.52% |
| C5d | C_5_r1 | 109,665,534 | 16,449,830,100 | 60.49% | 96.67% | 92.29% |
|  | C_5_r2 | 93,155,890 | 13,973,383,500 | 60.26% | 95.61% | 89.76% |
|  | C_5_r3 | 83,308,448 | 12,496,267,200 | 59.98% | 95.96% | 90.48% |
| C3d | C_3_r1 | 82,271,478 | 12,340,721,700 | 60.11% | 96.11% | 90.82% |
|  | C_3_r2 | 85,417,812 | 12,812,671,800 | 59.83% | 96.66% | 92.01% |
|  | C_3_r3 | 92,750,238 | 13,912,535,700 | 60.13% | 97.01% | 93.02% |
| T9d | T-9_r1 | 82,128,488 | 12,319,273,200 | 60.07% | 96.26% | 91.01% |
|  | T-9_r2 | 76,520,248 | 11,478,037,200 | 60.30% | 94.51% | 87.56% |
|  | T-9_r3 | 91,676,842 | 13,751,526,300 | 56.83% | 96.18% | 90.83% |
| T7d | T-7_r1 | 75,864,106 | 11,379,615,900 | 60.06% | 96.38% | 91.33% |
|  | T-7_r2 | 77,804,744 | 11,670,711,600 | 60.01% | 96.19% | 90.93% |
|  | T-7_r3 | 66,379,212 | 9,956,881,800 | 60.42% | 96.82% | 92.32% |
| T5d | T-5_r1 | 82,685,654 | 12,402,848,100 | 60.07% | 96.27% | 91.14% |
|  | T-5_r2 | 83,617,610 | 12,542,641,500 | 60.47% | 97.05% | 92.83% |
|  | T-5_r3 | 65,585,916 | 9,837,887,400 | 60.15% | 95.98% | 90.44% |
| T3d | T-3_r1 | 118,223,716 | 17,733,557,400 | 59.75% | 96.96% | 92.55% |
|  | T-3_r2 | 109,416,042 | 16,412,406,300 | 60.52% | 96.38% | 91.34% |
|  | T-3_r3 | 75,262,158 | 11,289,323,700 | 60.02% | 95.86% | 90.21% |
