# Supplementary material for: Multi-omics Analyses Provide Insight into the Biosynthesis Pathways of Fucoxanthin in Isochrysis galbana
Source: Genomics Proteomics Bioinformatics. 2022 Aug 13;20(6):1138–53. doi: 10.1016/j.gpb.2022.05.010 (PMC10225490; doi:10.1016/j.gpb.2022.05.010)
Supplement: Supplementary Table S23 — Mapping data of each transcriptome sample to the generated genome assembly [file mmc23.docx]

**Table S23 Mapping data of each transcriptome sample to the generated genome assembly**

| **Sample** | **Repeat** | **Total_reads** | **Unique_mapped** | **Multiple_mapped** | **Unmapped** |
| --- | --- | --- | --- | --- | --- |
| C9d | C_9_r1 | 33910551 | 93.62 | 1.54 | 4.84 |
|  | C_9_r2 | 37104165 | 93.45 | 1.52 | 5.03 |
|  | C_9_r3 | 41848238 | 93.41 | 1.5 | 5.09 |
| C7d | C_7_r1 | 54441171 | 92.25 | 1.79 | 5.96 |
|  | C_7_r2 | 45014556 | 92.68 | 1.5 | 5.82 |
|  | C_7_r3 | 37715476 | 93.38 | 1.77 | 4.85 |
| C5d | C_5_r1 | 54832767 | 92.76 | 1.7 | 5.54 |
|  | C_5_r2 | 46577945 | 93.34 | 1.68 | 4.98 |
|  | C_5_r3 | 41654224 | 92.6 | 1.93 | 5.47 |
| C3d | C_3_r1 | 41135739 | 93.56 | 1.42 | 5.02 |
|  | C_3_r2 | 42708906 | 93.48 | 1.45 | 5.07 |
|  | C_3_r3 | 46375119 | 93.63 | 1.61 | 4.76 |
| T9d | T_9_r1 | 41064244 | 93.68 | 1.64 | 4.68 |
|  | T_9_r2 | 38260124 | 92.53 | 1.65 | 5.82 |
|  | T_9_r3 | 45838421 | 91.11 | 1.84 | 7.05 |
| T7d | T_7_r1 | 37932053 | 93.96 | 1.61 | 4.43 |
|  | T_7_r2 | 38902372 | 93.37 | 1.59 | 5.04 |
|  | T_7_r3 | 33189606 | 93.24 | 1.81 | 4.95 |
| T5d | T_5_r1 | 41342827 | 93.83 | 1.52 | 4.65 |
|  | T_5_r2 | 41808805 | 93.61 | 1.68 | 4.71 |
|  | T_5_r3 | 32792958 | 93.35 | 1.6 | 5.05 |
| T3d | T_3_r1 | 59111858 | 93.65 | 1.72 | 4.63 |
|  | T_3_r2 | 54708021 | 93.16 | 1.65 | 5.19 |
|  | T_3_r3 | 37631079 | 93.47 | 1.45 | 5.08 |
